# *PNPLA3* p.I148M and *TM6SF2* p.E167K variants do not predispose to liver injury in cholestatic liver diseases: A prospective analysis of 178 patients with PSC

**DOI:** 10.1371/journal.pone.0202942

**Published:** 2018-08-30

**Authors:** Beata Kruk, Roman Liebe, Małgorzata Milkiewicz, Ewa Wunsch, Joanna Raszeja-Wyszomirska, Frank Lammert, Piotr Milkiewicz, Marcin Krawczyk

**Affiliations:** 1 Laboratory of Metabolic Liver Diseases, Center for Preclinical Research, Department of General, Transplant and Liver Surgery, Medical University of Warsaw, Warsaw, Poland; 2 Department of Medicine II, Saarland University Medical Center, Saarland University, Homburg, Germany; 3 Department of Medical Biology, Pomeranian Medical University in Szczecin, Szczecin, Poland; 4 Translational Medicine Group, Pomeranian Medical University in Szczecin, Szczecin, Poland; 5 Liver and Internal Medicine Unit, Medical University of Warsaw, Warsaw, Poland; University of Navarra School of Medicine and Center for Applied Medical Research (CIMA), SPAIN

## Abstract

**Background:**

The adiponutrin (*PNPLA3*) p.I148M and transmembrane 6 superfamily member 2 (*TM6SF2*) p.E167K variants represent major genetic risk factors for progressive liver injury in nonalcoholic fatty liver disease (NAFLD), alcoholic liver disease (ALD) and chronic viral hepatitis. The aim of this study was to find out whether these variants have a detrimental impact on the progression of chronic liver disease in patients with prolonged cholestasis induced by primary sclerosing cholangitis (PSC).

**Methods:**

We prospectively recruited 178 PSC patients (112 male, age range 17–75 years, 55 with liver cirrhosis, 94 with ulcerative colitis, 48 transplanted during follow-up). *PNPLA3* rs738409 and *TM6SF2* rs58542926 polymorphisms were genotyped using dedicated TaqMan assays. Associations between genotypes, biochemical and clinical phenotypes were analyzed using contingency tables.

**Results:**

Allele and genotype distribution of both variants were consistent with Hardy-Weinberg equilibrium. No significant differences in the genotype distribution of *PNPLA3* (P = 0.90) or *TM6SF2* (P = 0.72) were observed between patients with cirrhosis and patients without cirrhosis. Serum liver enzyme activities were not modified by the presence of *PNPLA3* (ALT P = 0.88, AST P = 0.77) or *TM6SF2* (ALT P = 0.92, AST P = 0.49) risk variants. Increasing number of risk alleles had no impact on serum liver enzyme activities, as demonstrated by a separate analysis of patients carrying 0 (n = 99), 1 (n = 64), 2 (n = 12) or 3 (n = 3) risk alleles (P>0.05). No impact of *PNPLA3* or *TM6SF2* risk variants was detectable in patients with PSC and ulcerative colitis, and none of the variants increased the odds of transplantation.

**Conclusions:**

Neither *PNPLA3* nor *TM6SF2* polymorphisms seem to contribute significantly towards an increased risk for deterioration of liver function in patients with PSC. These results underscore the divergent mechanisms of liver damage in cholestatic conditions as compared to metabolic and viral liver diseases.

## Introduction

Primary sclerosing cholangitis (PSC) is a progressive cholestatic liver disease characterized by ongoing inflammation and destruction of intrahepatic and extrahepatic bile ducts [1], and resulting in chronic liver damage leading to hepatic fibrosis. The precise etiology of PSC is unknown, but it is generally considered an immune-mediated disease [1]. As in other chronic liver diseases, the individual rates of disease progression vary substantially among patients, suggesting a contributory role for genetic modifiers of liver scarring in PSC.

Over the last decade, the genetic polymorphisms *PNPLA3* p.I148M (rs738409) and *TM6SF2* p.E167K (rs58542926) have been shown to be associated with an increased degree of liver injury in several chronic liver diseases. The *PNPLA3* "M" variant has been associated with increased hepatic steatosis and fibrosis in patients with viral hepatitis (both HCV and HBV), non-alcoholic fatty liver disease (NAFLD) [2] and alcoholic liver disease (ALD) [3], [4], [5], [6]. The *TM6SF2* "K" variant has so far been related to progression of liver disease in NAFLD, ALD and in HCV [7], [8], [9]. Both common modifier genes for the degree of liver damage in various aetiologies have not been intensely investigated in patients with PSC to date. Friedrich et al [10] studied 121 German and 347 Norwegian PSC patients and postulated that the disease-associated minor *PNPLA3* allele modifies the course of the disease among carriers of dominant stenosis in bile ducts. They found that risk allele carriers were more prone to require liver transplantation and had shorter survival. The impact of this variant on survival was restricted to males [10]. Italian researchers demonstrated recently that patients with inflammatory bowel disease (IBD) carrying the *PNPLA3* "M" allele are at increased risk of developing hepatic steatosis and increased liver enzymes [11]. They postulated that this variant might also modulate the degree of liver injury in patients with PSC and ulcerative colitis [11]. Of note, none of these studies investigated the *TM6SF2* polymorphism in the context of PSC.

Here we investigate if the *PNPLA3* p. I148M and *TM6SF2* p.E167K risk variants enhance liver injury in patients with chronic cholestasis. We hypothesized that these risk factors might synergise with inflammation-induced hepatic damage to increase disease progression. To verify our hypothesis, we studied the frequencies of both polymorphisms with respect to: 1) circulating markers of liver injury, 2) disease progression (evidence of liver cirrhosis, liver transplantation).

## Materials and methods

### Patients and methods

Prospectively we recruited patients of European descent with PSC at two Polish university centers (Warsaw and Szczecin). Informed consent was obtained from all patients, and the study protocol follows the ethical guidelines of the declaration of Helsinki as reflected in an a priori approval by the Research Ethics Committees of the Medical Universities of Warsaw and Szczecin. The diagnosis of PSC was based on the EASL criteria [12]. The presence of acute and chronic liver diseases other than PSC was excluded in all patients. IBD diagnosis was based on a complete colonoscopy with colonic biopsies. Blood samples were drawn for biochemical analyses and DNA genotyping. Liver enzyme activities (alkaline phosphatase (ALP), alanine transaminase (ALT), aspartate transaminase (AST), gamma-glutamyl transferase (GGTP) as well other biochemical blood tests were determined in the central laboratories of participating hospitals. Genotyping was performed in Department of Medical Biology (Szczecin, Poland). Diagnosis of liver cirrhosis was based on liver biopsy, clinical signs of liver cirrhosis, or imaging (i.e. transient elastography and/or magnetic resonance imaging). According to our previous study [13], the optimal transient elastography cut-off with maximum sensitivity and specificity to detect liver cirrhosis in PSC patients was determined as 13.7 kPa.

### Genotyping of the *PNPLA3* p.I148M and *TM6SF2* p.E167K polymorphisms

DNA for genotyping studies was extracted from blood samples using DNAasy Blood & Tissue Kit (Qiagen, Hilden, Germany). The *PNPLA3* (rs738409) and *TM6SF2* (rs58542926) variants were genotyped using TaqMan SNP Genotyping Assays (Assay ID C_7241_10, and Assay ID C_89463510_10, respectively; Applied Biosystems, Foster City, USA). Amplification conditions were as follows: 95°C for 10 min, 40 cycles of 92°C for 15 s, and 60°C for 1 min. The results were analyzed with allelic discrimination 7500 Software version 2.02.

### Statistics

All statistical analyses were performed using Statistica 12.0 (StatSoft, Poland) or GraphPad Prism 7.0 (GraphPad Software, CA, USA). Data were presented as medians (and ranges) for continuous variables and were analyzed using Student *t*-test or nonparametric Mann-Whitney U test or with ANOVA test, as appropriate. Associations between genotyped variants and tested variables were analyzed using contingency tables. Allele frequency differences were assessed by chi^2^ test and genotype differences by Armitage's trend test (https://ihg.gsf.de/cgi-bin/hw/hwa1.pl). The genotype frequencies of the *PNPLA3* and *TM6SF2* polymorphisms were tested for consistency with Hardy-Weinberg equilibrium (HWE) using exact tests.

## Results

### Clinical characteristics of study cohort

A total of 178 prospectively recruited patients with PSC (112 men, BMI range 15.7–32.0 kg/m^2^, 168 treated with UDCA 13‐15 mg/kg/day) were recruited between January 2012 and April 2016. Features of autoimmune hepatitis were present in 32 (17.9%) patients. None of the patients had small duct-PSC. Baseline characteristics of the analyzed cohort are summarized in Table 1 and in the S1 Table. In total, 114 (64%) had concomitant IBD: 94 (82.5%) ulcerative colitis, 7 (6.1%) Crohn`s disease and 13 (11.4%) presented with undifferentiated colitis. Cirrhosis was present in 55 patients. As shown in Table 1, the median age of the PSC was 35 (range 17–75) years, whereas median age at diagnosis of PSC was 31 (range 18–70) years. In patients with concomitant IBD the median age was 34 (range 17–75) years (P > 0.05 for comparison with patients without IBD).

**Table 1:** Clinical characteristics of study cohort.

Variables	Subject characteristics
N	178
Gender, female / male (%)	66 (37%) / 112 (63%)
Age (years)	35 (17–75)
Age at diagnosis (years)	31 (4–70)
IBD, none / UC / Crohn / undifferentiated colitis	64 / 94 / 7 / 13
Cirrhosis, (%)	55 (31%)
Liver transplantation, (%)	48 (26.9%)
BMI (kg/m^2^)	23.0 (15.7–32.0)

Values are given as medians and ranges.

Abbreviations: BMI, body mass index; IBD, inflammatory bowel disease; UC, ulcerative colitis.

### Effects of the *PNPLA3* p.I148M and *TM6SF2* p.E167K variants on liver injury in patients with PSC

**Fig 1:**
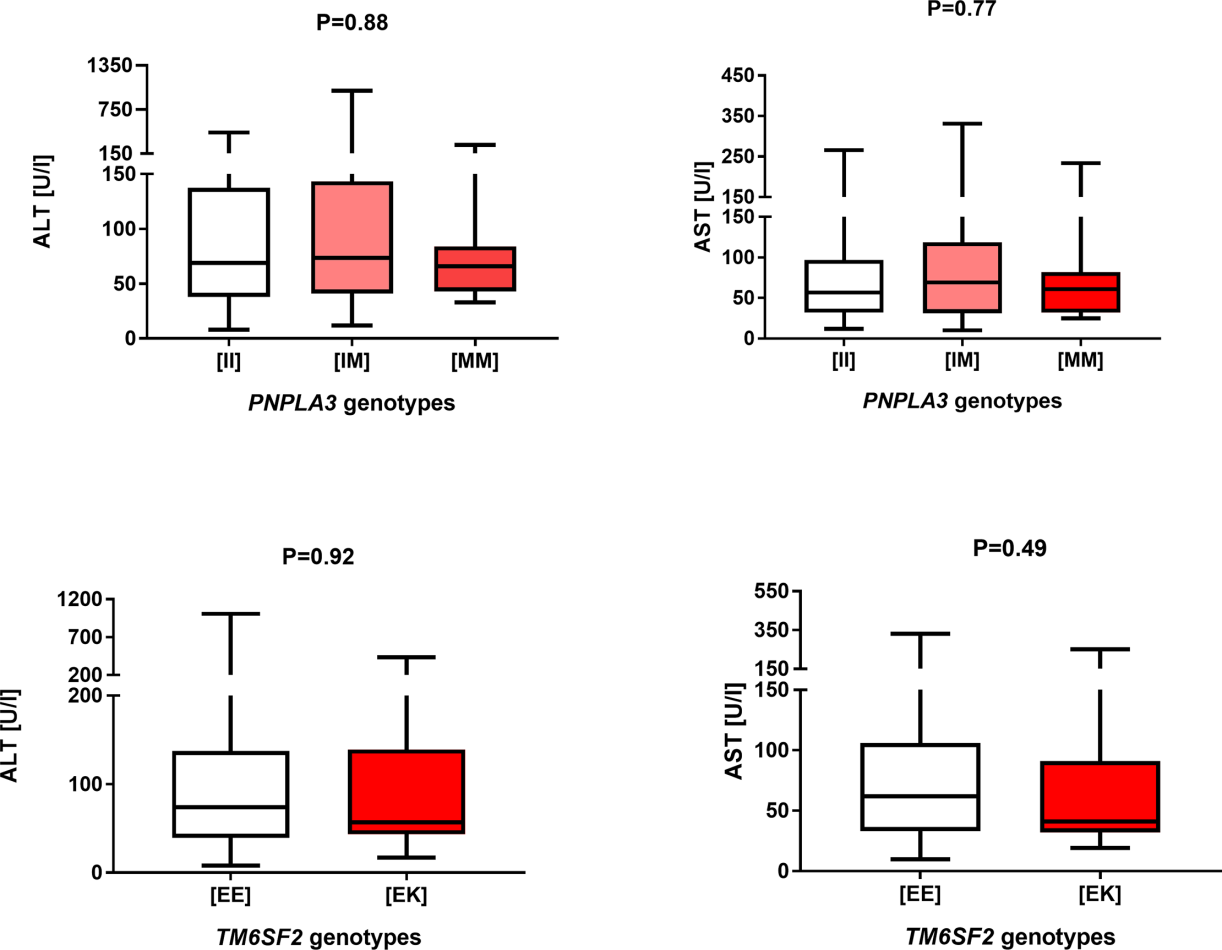
Box-and-whisker plots illustrating liver function tests in carriers of distinct *PNPLA3* and *TM6SF2* variants. We did not detect any major effects of these variants on the AST and ALT activities.

**Fig 2:**
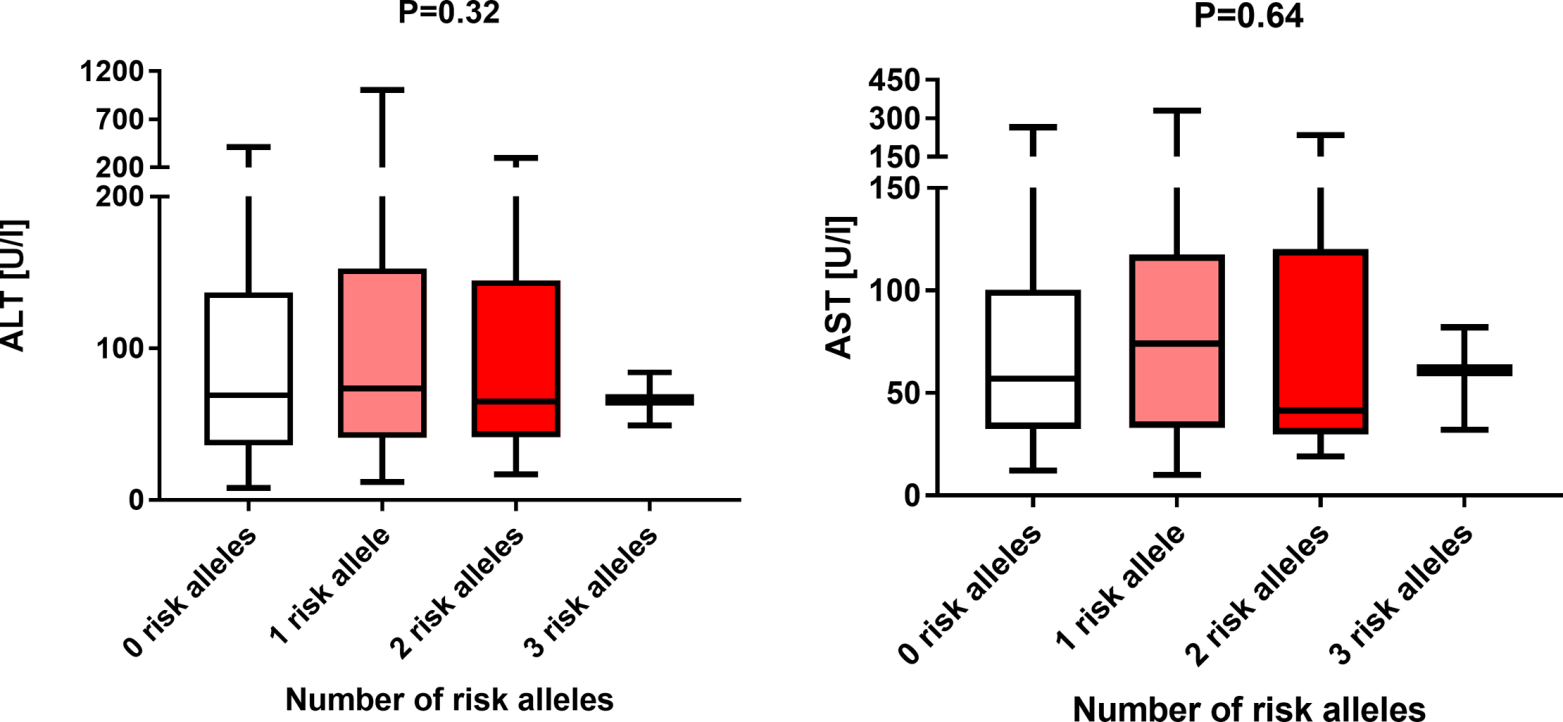
Combined analysis of the *PNPLA3* p.I148M and *TM6SF2* p.E167K risk alleles on liver function tests. The following frequencies of carriers of risk alleles were detected: zero risk alleles, n = 99; one risk allele, n = 64; two risk alleles, n = 12; three risk alleles, n = 3.

All included patients were successfully genotyped. Overall, we did not detect any deviation from the HWE for either the *PNPLA3* (P = 0.90) or *TM6SF2* (P = 0.72) genotypes. As presented in Table 2, the *PNPLA3* p.I148M minor allele was carried by 69 (38.8%) patients with PSC, and the *TM6SF2* minor allele was detected in 21 (11.8%) of patients. Of note, only 7 (4.0%) patients were homozygous carriers of the *PNPLA3* p.I148M allele, while we did not detect any homozygotes for the *TM6SF2* variant. Analysis of the association between the *PNPLA3* and *TM6SF2* variants and liver function tests is presented in Fig 1. As illustrated, none of the tested variants was associated with increased activities of AST or ALT (both P > 0.05). Liver function tests did not increase with increasing numbers of risk alleles as demonstrated by a separate analysis of patients carrying 0 (n = 99), 1 (n = 64) or 2 (n = 12) or 3 (n = 3) minor alleles (Fig 2; P > 0.05).

**Table 2:** Distribution of the *PNPLA3* and *TM6SF2* genotypes in PSC patients.

Genotypes	Count of genotypes
*PNPLA3* [II]	109 (61.2%)
*PNPLA3* [IM]	62 (34.8%)
*PNPLA3* [MM]	7 (4.0%)
*TM6SF2* [EE]	157 (88.2%)
*TM6SF2* [EK]	21 (11.8%)
*TM6SF2* [KK]	0 (0%)

Abbreviations: E, glutamic acid; I, isoleucine K; lysine; M, methionine; *PNPLA3*, patatin-like phospholipase domain-containing protein 3*TM6SF2*, transmembrane 6 superfamily member; PSC, primary sclerosing cholangitis

Subsequently, we compared the distribution of both variants in patients with and without liver cirrhosis (n = 55 and n = 123, respectively). Table 3 presents the frequencies of each variant in these groups of patients. After stratification, *PNPLA3* (P = 0.75) or *TM6SF2* (P = 0.62) genotype distributions did not differ between patients with cirrhosis and without cirrhosis.

**Table 3:** *PNPLA3* and *TM6SF2* genotypes in patients with and without liver cirrhosis.

Patients with liver cirrhosis				Patients without liver cirrhosis			
*PNPLA3* p.I148M		*TM6SF2* p.E167K		*PNPLA3* p.I148M		*TM6SF2* p.E167K	
[II] n = 33		[EE] n = 50		[II] n = 76		[EE] n = 107	
[IM] n = 19		[EK] n = 5		[IM] n = 43		[EK] n = 16	
[MM] n = 3		[KK] n = 0		[MM] n = 4		[KK] n = 0	

Abbreviations: see Table 2.

During follow-up, 48 (26.9%) patients underwent liver transplantation. The minor *PNPLA3* and *TM6SF2* alleles were carried by 23 and 7 of these individuals, respectively (Table 4), and did not differ from the frequencies in patients who did not require transplantation.

**Table 4:** *PNPLA3* and *TM6SF2* genotypes in patients who underwent and who did not require liver transplantation during follow-up.

Patients who underwent liver transplantation				Patients who did not require liver transplantation			
*PNPLA3* p.I148M		*TM6SF2* p.E167K		*PNPLA3* p.I148M		*TM6SF2* p.E167K	
[II] n = 25		[EE] n = 41		[II] n = 84		[EE] n = 116	
[IM] n = 20		[EK] n = 7		[IM] n = 42		[EK] n = 14	
[MM] n = 3		[KK] n = 0		[MM] n = 4		[KK] n = 0	

Abbreviations: see Table 2.

The effects of the *PNPLA3* (P = 0.38) or *TM6SF2* (P = 0.58) variants were also neither apparent in patients with nor in patients without ulcerative colitis (n = 55 and n = 123, respectively). Table 5 summarizes the frequencies of each variant in these groups. The findings of our study suggest that neither of the two polymorphisms affects the risk of cirrhosis (*PNPLA3* P = 0.27, *TM6SF2* P = 0.92) or the odds of requiring liver transplantation (*PNPLA3* P = 0.66, *TM6SF2* P = 0.77) in PSC patients with concomitant ulcerative colitis.

**Table 5:** *PNPLA3* and *TM6SF2* genotypes in PSC patients with and without ulcerative colitis.

Patients with ulcerative colitis				Patients without ulcerative colitis			
*PNPLA3* p.I148M		*TM6SF2* p.E167K		*PNPLA3* p.I148M		*TM6SF2* p.E167K	
[II] n = 67		[EE] n = 85		[II] n = 37		[EE] n = 53	
[IM] n = 26		[EK] n = 9		[IM] n = 23		[EK] n = 11	
[MM] n = 1		[KK] n = 0		[MM] n = 4		[KK] n = 0	

Abbreviations: see Table 2.

**Fig 3:**
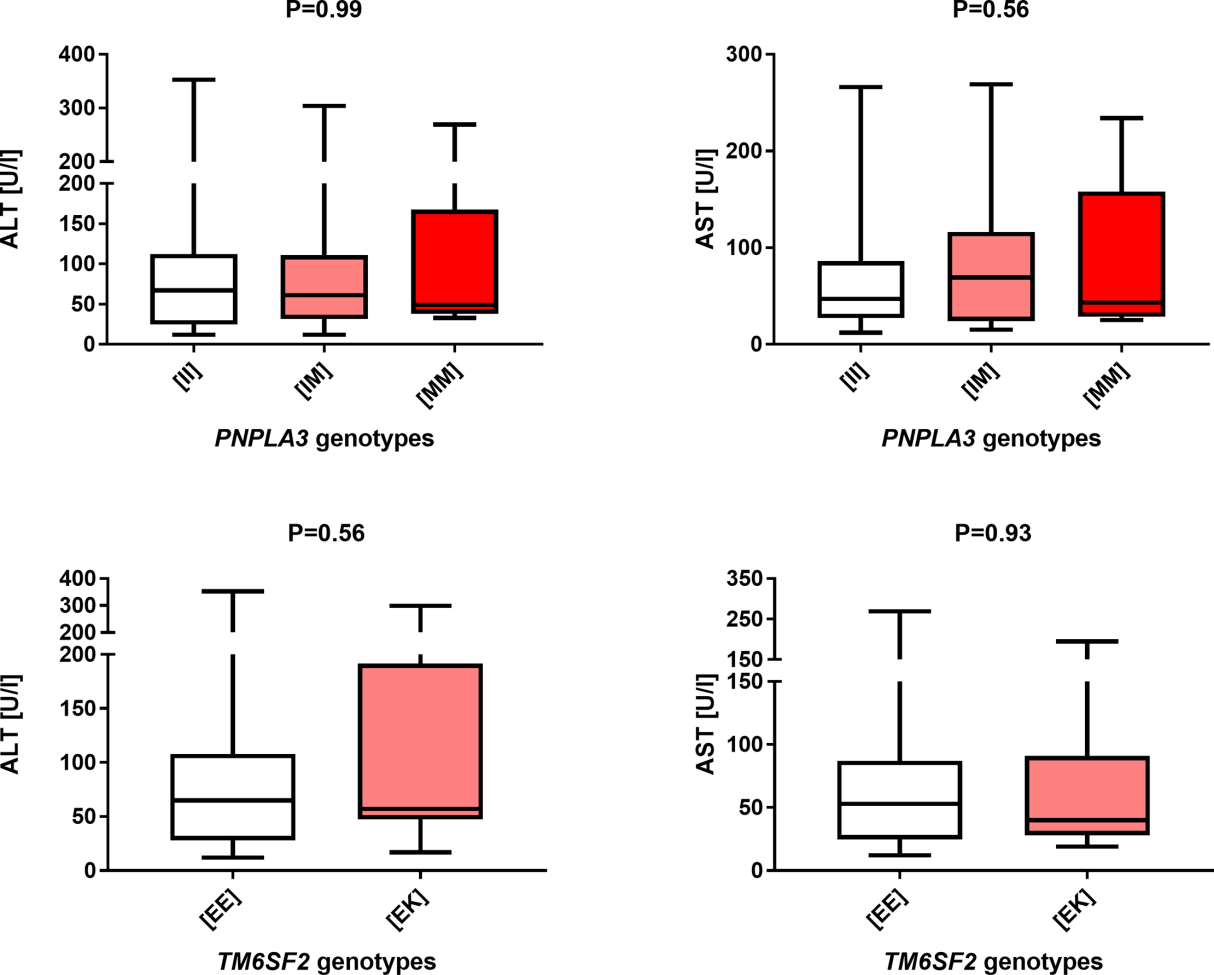
Box-and-whisker plots illustrating liver function tests in carriers of distinct *PNPLA3* and *TM6SF2* variants in females. We did not detect any major effects of these variants on the AST and ALT activities.

**Fig 4:**
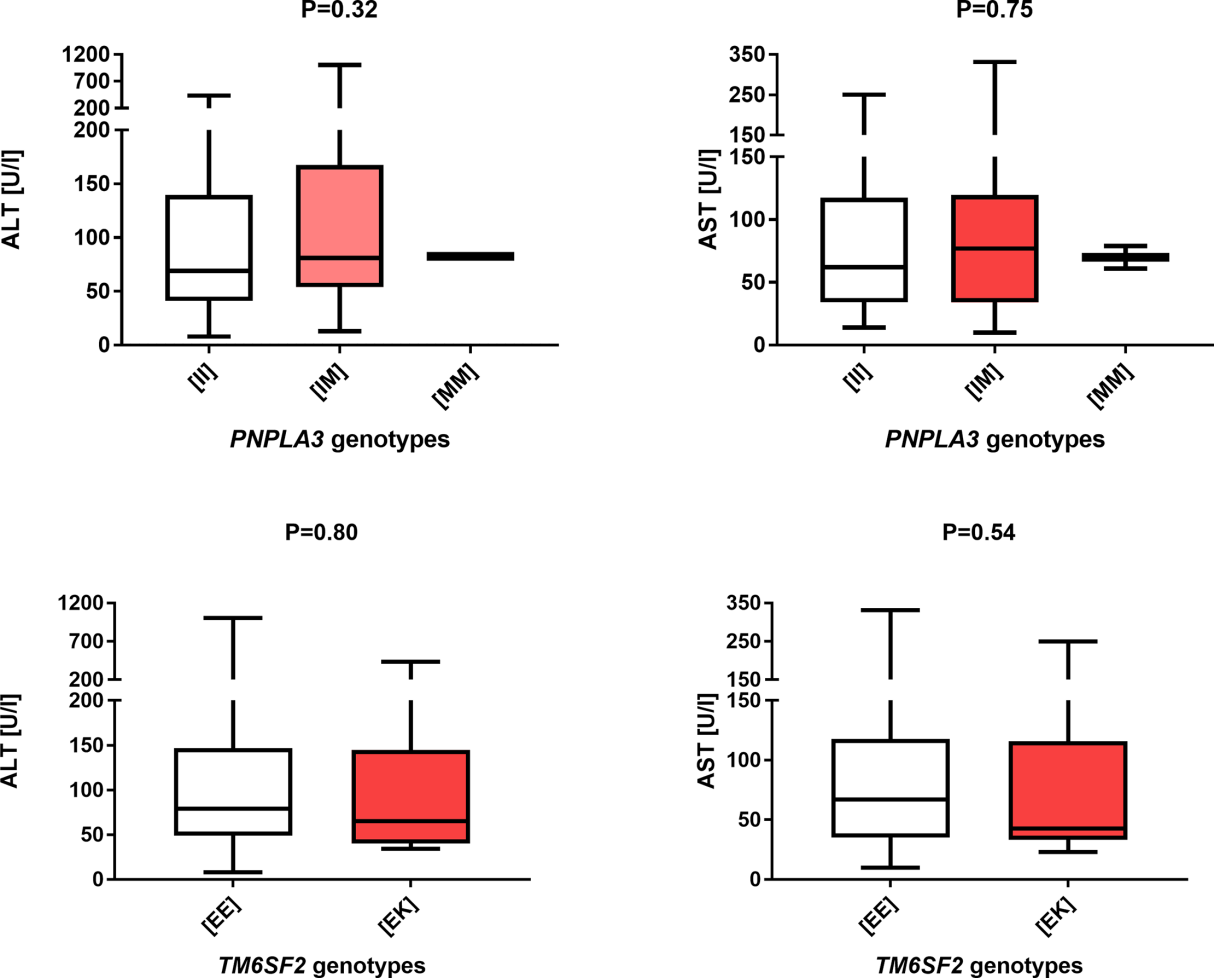
Box-and-whisker plots illustrating liver function test in carriers of distinct *PNPLA3* and *TM6SF2* variants in males. We did not detect any major effects of these variants on the AST and ALT activities.

Finally, we did not detect a sex-specific impact of the *PNPLA3* (female patients P = 0.57, male patients P = 0.84) or *TM6SF2* polymorphisms (female patients P = 0.91, male patients P = 0.72) on the frequency of cirrhosis. As shown in Figs 3 and 4, subgroup analysis of the association between *PNPLA3* and *TM6SF2* variants and liver function tests in women and men did not reach significance (all P > 0.05). The analysis showed no impact of the polymorphisms on liver transplantation in either gender (women: *PNPLA3* P = 0.94, *TM6SF2* P = 0.69; men: *PNPLA3* P = 0.59, *TM6SF2* P = 0.70). Of note, also after exclusion of patients with potential overlap with AIH, none of the above tests proved any association between tested variants and liver injury or diseases progression (all P > 0.05).

## Discussion

Considering the profound impact of the *PNPLA3* p.I148M and *TM6SF2* p.E167K variants on the progression of chronic liver diseases of various aetiologies, one might expect an impact in the setting of PSC. Mechanistically, the hepatotoxic effect of reactive lipid species conveyed by the risk variants of both genes might enhance the inflammatory damage inflicted by PSC. Our study indicates that in chronic cholestasis, neither *PNPLA3* nor *TM6SF2* polymorphisms seem to confer a significant additional risk for the deterioration of liver function.

PSC is an infrequent condition. The latest available data show that this disease affects less than 250,000 adults in Europe [14]. Hence, the recruitment of larger cohorts, which provide the necessary statistical power to identify genetic modifiers of the disease, is rather difficult. In the current study, we prospectively recruited a sizeable cohort including information on detailed hepatic phenotypes required to detect genetic modifiers of liver injury [15]. In the past, a common physiological core pathway of fibrogenesis has been suggested [16]. Considering the various possible sources of hepatic injury represents an invitation to speculate about possible additive or synergistic effects of autoimmune inflammation and lipid overload. However, our results demonstrate that the liver damage in cholestatic liver conditions appears to be due to different and non-additive mechanisms compared to viral or metabolic liver diseases. Thus, while the *PNPLA3* and *TM6SF2* risk alleles enhance the damage exerted by viral activity or metabolic overload, their impact on hepatocellular lipid content does not seem to compound the damage caused by inflamed bile ducts.

In our previous study [17] we demonstrated by transient elastography that the *PNPLA3* polymorphism might be associated with increased liver stiffness, a surrogate marker of liver fibrosis, in patients with chronic liver diseases. Results from studies in large cohorts [18], [2] underscored that the *PNPLA3* p.I148 variant plays an important role in the progression from steatosis to fibrosis and cirrhosis. Other investigations [5], [19] revealed that carriers of the *PNPLA3* risk allele have increased serum ALT activities. Nevertheless, in the current study we did not detect any significant associations of either the *PNPLA3* or the *TM6SF2* polymorphism with any signs of liver injury in the setting of PSC. This observation is in line with Sookoian et al. [20] who did not find any association between variant *TM6SF2* and liver function tests in patients with NAFLD. On the other hand, we showed in our previous analysis that Germans with fatty liver carrying the minor *TM6SF2* allele have increased liver function tests [2]. These discrepancies are quite likely due to the low frequency of the *TM6SF2* minor allele (i.e. <1%). Based on our previous studies [18], [21] concerning both variants, also in Polish patients [22], we reckon that our cohort in the present study was powered to detect potential associations between the *PNPLA3* variant and liver injury in patients with PSC. Due to its relative rarity, larger cohorts might be required to investigate the impact of the *TM6SF2* risk allele.

Compared to other chronic liver diseases, little is known about genetic modifiers of liver injury in cholestatic conditions. Alberts et al [23] analyzed 3,402 patients with PSC who were genotyped using an “Immunochip” (variants n = 130,422). The authors identified association between rs853974 in the gene *RSPO3* and transplant-fee survival, and this variant also influenced the risk of PSC-related death during the follow-up [23]. In our study, we used a candidate-gene study design focusing on selected variants previously associated with liver injury. Although we availed of an extensive and well-characterized cohort of patients with PSC, it is possible that it was underpowered to detect more subtle modifier effects of *PNPLA3*. Friedrich et al [10] demonstrated in 121 German PSC patients that presence of the p.I148M risk allele was associated with reduced survival in those who had dominant stenosis; this effect was the most pronounced in males. This observation was subsequently validated in a cohort of 347 Norwegian individuals with PSC [10]. In our cohort we did not detect any sex-specific effects of *PNPLA3*. However, since organ allocation guidelines differ between Germany, Norway and Poland, it is not straightforward to compare survival rates in cohorts from these different countries.

Overall, based on our results we conclude that neither the *PNPLA3* p.I148M nor the *TM6SF2* p.E167K variant are major contributors towards hepatocellular damage or fibrosis progression in patients with PSC. Further mechanistic and cohort studies will be required to clarify the potential influence of these or other variants on the progression of cholestatic liver diseases.

## Supporting information

S1 Table Laboratory characteristics of the study cohort. (DOCX)
