# Supplementary material for: PNPLA3 p.I148M and TM6SF2 p.E167K variants do not predispose to liver injury in cholestatic liver diseases: A prospective analysis of 178 patients with PSC
Source: PLoS One. 2018 Aug 30;13(8):e0202942. doi: 10.1371/journal.pone.0202942 (PMC6117000; doi:10.1371/journal.pone.0202942)
Supplement: S1 Table — (DOCX) [file pone.0202942.s001.docx]

**S1 Table.**

**Laboratory characteristics of the study cohort.**

| **Variables** | **Subject characteristics** |
| --- | --- |
| Hemoglobin (mg/dl) | 13.0 (7.8 – 17.4) |
| PLT (10^3^/µl) | 223 (42 – 749) |
| Creatinine (mg/dl) | 0.7 (0.25 – 2.5) |
| Bilirubin (mg/dl) | 1.2 (0.18 – 33.0) |
| ALT (U/L) | 66 (8 – 1005) |
| AST (U/L) | 57 (10 – 709) |
| ALP (U/L) | 238 (41 – 1360) |
| GGTP (U/L) | 175 (7 – 1758) |
| Albumin (mg/dl) | 4.0 (2.3 – 5.2) |
| INR | 1.1 (0.8 – 4.0) |

Values are given as medians and ranges.

Abbreviations: ALP, alkaline phosphatase; ALT, alanine transaminase; AST, aspartate transaminase; GGTP, gamma-glutamyl transferase; INR, international normalized ratio; PLT, platelet count.
